# 

**Published:** 1906-10

**Authors:** 


Vol. XXII. OCTOBER, 1906. No. 4.

OCT. 15

### TEXAS MEDICAL JOURNAL

"RED BACK"

PUBLISHED AT AUSTIN, TEXAS, BY
F. E. DANIEL, M. D., - - Proprietor
PRINTED BY THE VON BOECKMANN-JONES CO.

**Subscription, $1.00 a Year, in Advance. - - - Single Copies, 10 Cents**

Entered at the Postoffice at Austin, Texas, as Second-class Mail Matter

